# Multicomponent non-pharmacological interventions for preventing delirium in critically ill patients: a systematic review and meta-analysis of randomized controlled trials

**DOI:** 10.3389/fnins.2026.1927572

**Published:** 2026-09-24

**Authors:** Shiting Wang, Jiamin Lei, Wenjing Huang, Huajian Chen, Shufang Li

**Affiliations:** 1 The Eighth Clinical Medical College of Guangzhou University of Chinese Medicine, Foshan, Guangdong, China; 2 Foshan Hospital of Traditional Chinese Medicine, Foshan, Guangdong, China

**Keywords:** delirium, intensive care unit, meta-analysis, multicomponent non-pharmacological interventions, systematic review

## Abstract

**Background:**

It is necessary to evaluate the efficacy of multicomponent non-pharmacological interventions in preventing and managing intensive care unit (ICU) delirium and identify the most effective intervention combinations.

**Methods:**

A systematic literature search was conducted in PubMed, EMBASE, CINAHL, Cochrane Library, and ProQuest Dissertations and Theses A&I databases from inception to July 3, 2026, supplemented by a hand search of prior meta-analyses. Only randomized trials were included. Meta-analysis was performed using risk ratios (RR) or mean differences (MD) with 95% confidence intervals (CI). Random-effects models with restricted maximum likelihood estimation and modified Hartung-Knapp confidence intervals were used. Risk of bias was assessed using the Cochrane risk-of-bias tool for randomized controlled trials. The certainty of evidence was assessed using the GRADE approach.

**Results:**

The search identified 13 randomized controlled trials encompassing 5,525 patients. Multicomponent interventions significantly reduced delirium incidence (RR 0.53, 95% CI [0.36, 0.76], I^2^ = 72.5%, low-certainty evidence). The effect remained significant after excluding the two cluster-randomized trials (RR 0.45, 95% CI 0.30–0.67; I^2^ = 55.7%), the active-control trial (RR 0.55, 95% CI 0.38–0.79; I^2^ = 73.0%), and all three trials simultaneously (RR 0.46, 95% CI 0.31–0.70; I^2^ = 57.0%). In exploratory subgroup analysis, the within-subgroup pooled estimate for component pattern B, comprising cognitive assessment and stimulation, reorientation, sleep and environmental management, family or community support, and physical activity, showed a reduced incidence of delirium (RR 0.43, 95% CI [0.23, 0.82]); however, the formal test for subgroup differences was not significant (*p* = 0.604). No statistically significant effects were found for delirium duration, ICU or hospital length of stay, delirium-free days, or mortality.

**Conclusion:**

This study demonstrated that multi-component interventions effectively reduce delirium incidence in critically ill patients. The evidence did not establish that one component combination was superior to another.

**Systematic review registration:**

https://www.crd.york.ac.uk/PROSPERO/view/CRD42024592821.

## 1 Introduction

Delirium is an acute-onset and fluctuating brain dysfunction characterized by impaired attention and awareness, and is a common complication among patients admitted to the intensive care unit (ICU) (Devlin et al., 2018; Kasapoğlu and Enç, 2022). While the incidence of delirium in hospitalized patients is approximately 20–30% (Siddiqi et al., 2016), it increases significantly to 25–87% among ICU patients, depending on illness severity and diagnostic methods (Girard et al., 2010; Kang et al., 2018; Morandi et al., 2009; Pandharipande et al., 2008). ICU delirium is associated with various adverse outcomes, including prolonged mechanical ventilation (Jeon et al., 2016), long-term cognitive decline (Goldberg et al., 2020), extended ICU and hospital stays (Salluh et al., 2015), and increased workload for clinical nurses (Öztürk Birge and Bedük, 2018; Parry et al., 2018; Yue et al., 2015). These risks highlight the necessity of implementing early preventive measures to improve prognoses in critically ill patients.

Pharmacologic interventions, such as dexmedetomidine, atypical antipsychotics, and haloperidol, are commonly used for ICU delirium prevention and management due to their rapid onset, widespread availability, and potential to reduce hospital length of stay (Burry et al., 2021; León-Salas et al., 2020; Mu et al., 2015). However, these treatments may carry significant risks, including arrhythmias and respiratory distress, particularly in vulnerable patients (Ice et al., 2016; Wang et al., 2020; Yagmur et al., 2010). Additionally, studies have demonstrated limited efficacy of pharmacological interventions in reducing delirium incidence (Zitikyte et al., 2023). Bannon’s study state that insufficient evident to support single or multicomponent non-pharmacological interventions in reducing delirium rate or duration, but multi-component interventions may present a more credible opportunity to target several risk factors (Bannon et al., 2019). Current PADIS guidelines and recent research advocate for multicomponent non-pharmacological strategies as the most effective approach to prevent and manage ICU delirium (Devlin et al., 2018; Gómez Tovar and Henao Castaño, 2024; Lange et al., 2022).

To date, multiple meta-analyses have evaluated the effectiveness of single-component and multicomponent non-pharmacological interventions in preventing ICU delirium (Kang et al., 2018; Chen et al., 2022; Deng et al., 2020; Matsuura et al., 2023). These studies consistently demonstrated that multicomponent non-pharmacological interventions significantly reduce the incidence of delirium. However, their effectiveness in shortening delirium duration remains a topic of debate. While three studies (Kang et al., 2018; Chen et al., 2022; Deng et al., 2020) reported no significant impact of multi-component interventions on delirium duration, Chen’s et al. (2022) study suggested that specific intervention combinations could achieve a significant reduction.

Matsuura et al. (2023) identified two effective multi-component intervention combinations for delirium prevention: (1) sleep promotion, cognitive stimulation, early activity, pain control, and assessment, and (2) sleep promotion and cognitive stimulation. However, their conclusions were based on a combined analysis of studies with varying designs, which may have resulted in inaccurate estimates of the comparative effectiveness of non-pharmacological strategies in reducing ICU delirium incidence. Given the availability of numerous randomized controlled trials (RCTs), an updated systematic review and meta-analysis focusing exclusively on RCTs is clinically relevant.

Moreover, no study has systematically defined the specific implementation details of these interventions. A key innovation of this study is the comprehensive listing and integration of the implementation protocols for various interventions, providing precise and actionable guidance for clinical practice.

This study aimed to conduct an updated systematic review and meta-analysis to evaluate the efficacy of multicomponent non-pharmacological interventions in preventing and managing ICU delirium, identify the most effective intervention combinations, address existing controversies regarding their impact on delirium duration and ICU stay, and offer evidence-based recommendations to enhance clinical practice.

## 2 Methods

### 2.1 Study design and protocol

This systematic review and meta-analysis adhered to the Preferred Reporting Items for Systematic Reviews and Meta-Analyses (PRISMA) guidelines and was registered with the International Prospective Register of Systematic Reviews (PROSPERO: CRD42024592821) (Page et al., 2020).

### 2.2 Eligibility criteria (PICOS)

We included studies based on the following criteria: (1) Types of studies: randomized controlled trials (RCTs); (2) Participants: ICU adult patients; (3) using multicomponent non-pharmacological interventions as the intervention group; (4) using either non-pharmacological interventions or routine care as the control group; (5) Research content: multicomponent non-pharmacological interventions prevention and treatment delirium.

Studies were excluded based on the following criteria: (1) Reviews, letters, abstracts, conference proceedings, duplicate publications, or articles without full-text availability. (2) Non-English studies. (3) Studies involving neonatal or pediatric ICU patients. (4) Use of pharmacological interventions or single non-pharmacological interventions.

### 2.3 Search strategy and data sources

A comprehensive search was conducted across five electronic databases: PubMed, EMBASE, CINAHL, Cochrane Library, and ProQuest Dissertations and Theses A&I, covering studies from inception to July 3, 2026. The search used the following keyword combinations: (“critical care” OR “intensive care” OR “ICU”) AND (“non-pharmacologic” OR “non-pharmacological” OR “multicomponent” OR “bundle” OR “mobilization” OR “daily sedation interruption” OR “music” OR “light therapy” OR “reorientation” OR “cognitive stimulation” OR “cognitive training” OR “eye mask” OR “earplugs” OR “physical therapy” OR “occupational therapy” OR “multicomponent”) AND delirium. Additionally, the reference lists of relevant studies were manually reviewed to identify eligible studies for inclusion in this review.

### 2.4 Study selection and data extraction

Two researchers (Wang ST and Huang WJ) independently conducted the literature screening and data extraction. Any disagreements were resolved through discussion or consultation with a third researcher, if necessary. The screening process involved three steps: (1) reviewing titles and abstracts, (2) examining full texts, and (3) reviewing references of selected studies. At each step, the inclusion and exclusion criteria were applied consistently.

Relevant data were extracted from the included articles using a standardized form. The extracted items included the first author, year of publication, study region, study design, sample size, average age, setting, types of interventions, type of center, and other relevant details.

### 2.5 Intervention component classification

After data extraction, the intervention content of each included trial was reviewed in detail. Based on established non-pharmacological approaches to ICU delirium prevention and the actual intervention elements reported in the included studies, the interventions were summarized into five components: cognitive assessment and stimulation, reorientation, sleep and environmental management, family or community support, and physical activity. The specific combination of these components was then used to classify interventions into three component patterns, as shown in Tables 1, 2. This classification was used to support the exploratory subgroup analysis and should not be interpreted as an assessment of the independent effect of any single component.

**Table 1:** Classification of intervention component patterns used for exploratory subgroup analysis.

Component pattern	Component combination
Pattern A	Cognitive assessment and stimulation; reorientation; sleep and environmental management; family or community support
Pattern B	Cognitive assessment and stimulation; reorientation; sleep and environmental management; family or community support; physical activity
Pattern C	Cognitive assessment and stimulation; reorientation; sleep and environmental management; physical activity

**Table 2:** Components of non-pharmacological interventions.

Component-based strategies	Treatment-based strategies
① Cognitive assessment and stimulation	Use proven ICU delirium and sedation identification tools such as CAM-ICU, CAM, RASS etc. Intense external stimulation with an objective of increasing the patient’s level of alertness. Pool of exercises with an objective of activating mental functions, which include the following areas, among others: alertness, visual perception, memory, calculus, problem solving, praxis, and language. Each patient received a notebook for cognitive exercises, as well as tools such as sequencing cards and games (cards, dominoes and memory, and visuospatial construction games). Correction of sensory deficits, such as encouraging patients to use glasses and hearing aids, protects the cornea from dryness and cleans the ear canal. Encourage positive communication and use an appropriate communication technique (verbal/written/pictures) to compensate sensory loss and overcome language barriers. Allow patients watch TV and listen to preferred music or read a newspaper/magazine when possible. Daily spontaneous awakening trial (SAT) or Daily spontaneous breathing trial (SBT) was given for the patient with mechanical ventilated.
② Reorientation	Provide visible clock, calendar, cell phone, and schedule for patients. Inform the department, the hospital and the reason for admission.
③ Management of sleep and environmental	Specific measures to maintain a good sleep–wake cycle were adopted. For example, the room was illuminated by natural light during the day and by downy light at night. All nursing procedures were minimized in the night. Noise was decreased as much as possible. For example, various alert tones of the alarm system were modulated to be gentler. Provide an eyeshade and acoustic earplugs for patients in sleeping to a good sleep. Limited movement to other beds, individualized their bedside area, and allow to bring home favorites (pillow/family photo). The staff invited the patients to visit the ICU to become acquainted with the environment.
④ Support of family	Extend family visits as long as possible and encourage family involvement in the patients’ daily care. Use effective communication devices to talk to family members. Identify patients’ needs and possible causes of stress and address them with the help of family members.
⑤ Physical activity	Encourage early mobilization and plan mobility schedule. Basic activities of daily living (BADLs), group of activities that promotes independent living based on BADL that include hygiene, personal grooming, and eating. Mobilize the patient each shift. If −2 to −4 RASS fowler position, massage, exercise on bed, position changes each 2 h, moving joints pedaling. If −1 to 2 RASS, fowler position, chairs sitting, standing, small steps with help. Minimizing and optimally locating restraining lines.

### 2.6 Risk-of-bias assessment

The methodological quality of the included studies was assessed independently by two reviewers (Wang ST and Huang WJ) using the Cochrane risk-of-bias tool for randomized controlled trials (Higgins et al., 2011). Risk of bias was evaluated across seven domains: random sequence generation, allocation concealment, blinding of participants and personnel, blinding of outcome assessment, incomplete outcome data, selective reporting, and other potential sources of bias. Each domain was judged as low, unclear, or high risk of bias. Disagreements were resolved through discussion or consultation with a third reviewer.

### 2.7 Certainty of evidence

The certainty of evidence for each outcome was assessed using the Grading of Recommendations Assessment, Development, and Evaluation (GRADE) approach (Guyatt et al., 2011). As all included studies were randomized controlled trials, the certainty of evidence was initially rated as high and then downgraded, where appropriate, for risk of bias, inconsistency, indirectness, imprecision, and publication bias. The final certainty of evidence was classified as high, moderate, low, or very low.

### 2.8 Data analysis

Data were extracted from the original studies, and the updated meta-analysis was conducted using R version 4.4.2. Risk ratios (RRs) were used for dichotomous outcomes, and mean differences (MDs) for continuous outcomes. Data reported in formats other than means and standard deviations were converted only when clinically and statistically appropriate. Effect estimates are presented with 95% confidence intervals (CIs). Random-effects meta-analyses were performed using restricted maximum likelihood estimation and the modified Hartung–Knapp method. Heterogeneity was assessed using the χ^2^ test and quantified using the I^2^ statistic. For the two cluster-randomized trials, effect estimates adjusted for clustering were used. One trial reported an adjusted odds ratio, which was converted to an approximate risk ratio using the observed event risk in the control group; its standard error was estimated using the delta method. The robustness of the primary analysis was examined by separately excluding the two cluster-randomized trials and the active-control trial, followed by an analysis excluding all three trials simultaneously. Potential publication bias and small-study effects for delirium incidence were assessed through visual inspection of the funnel plot and Egger’s regression test.

## 3 Results

### 3.1 Study selection

A total of 13,086 articles were identified through electronic database searches, and an additional four articles were retrieved through hand-searching strategies. After the removal of 156 duplicate records and marked 62 ineligible Records by automation tools, the titles and abstracts of 12,868 articles were screened, resulting in the exclusion of 12,831 articles that did not meet the inclusion criteria. The remaining 37 full-text articles were assessed for eligibility, with 24 excluded due to reasons such as inclusion of non-ICU patients, non-randomized controlled trials, non-multicomponent non-pharmacological interventions, or failure to report the incidence of delirium. After reviewing reference lists of the eligible studies, 13 articles met the criteria and were included in the systematic review (see Figure 1).

**Figure 1:**
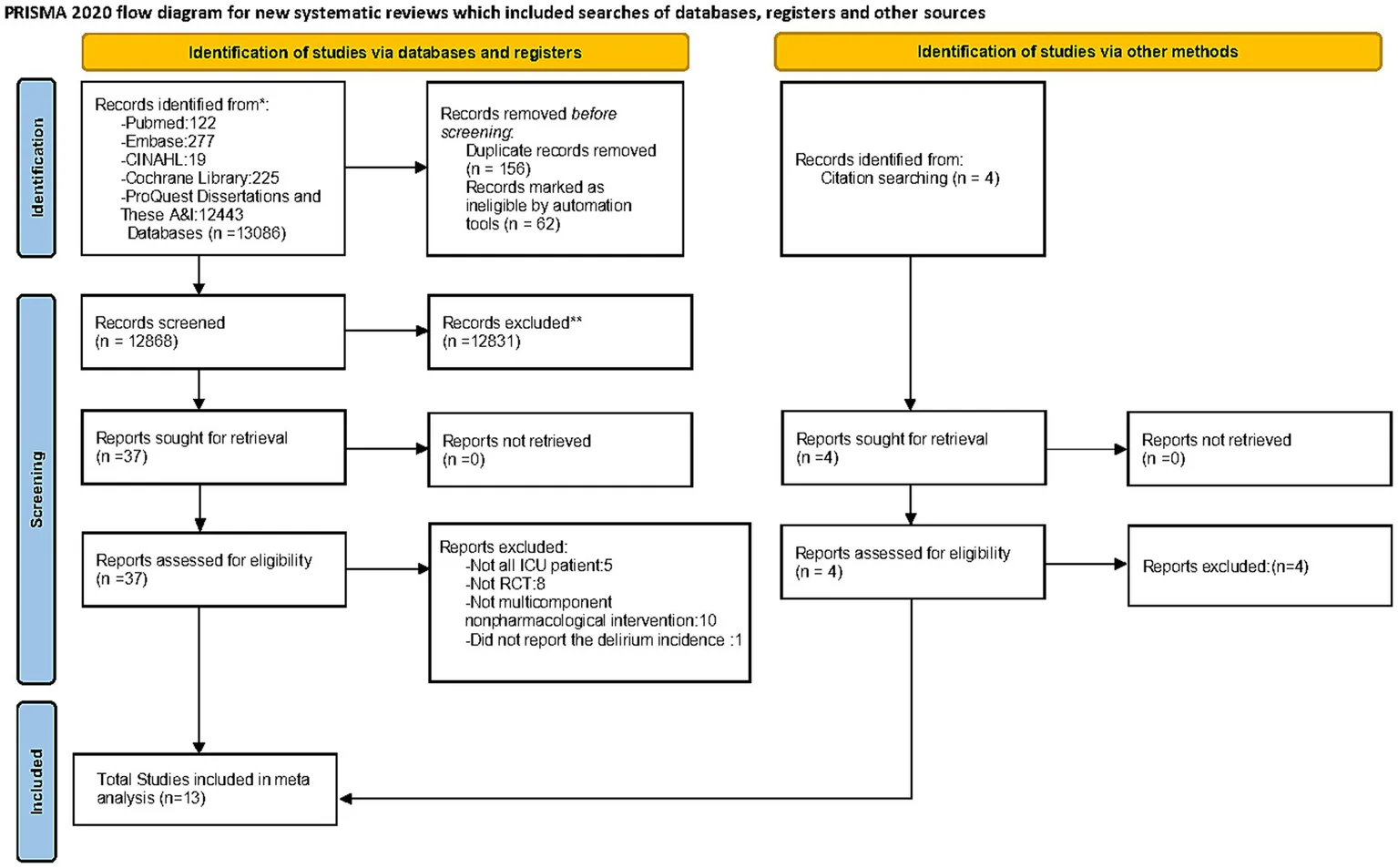
PRISMA 2020 flow diagram for multicomponent non-pharmacological interventions comparison in reducing ICU delirium.

### 3.2 Characteristics of the included studies

The review incorporated 13 randomized controlled trials, including two stepped-wedge cluster randomized trials, with sample sizes ranging from 40 to 2,618 participants, totaling 5,525 individuals. These studies were published from 2015 to 2026. All studies reported data on delirium incidence. The basic characteristics of the included studies are detailed in Table 3.

**Table 3:** Characteristics of studies.

Study	Country	Study design	Setting	Multicenter	Sample (*N*) Total-I-C	Mean patient Age, y	Delirium assessment	Type of interventions
Gómez Tovar and Henao Castaño (2024)	Colombia	RCT	ICU	N	213-71-142	60.2	CAM-ICU	①②③④⑤
Brennan et al. (2023)	Australia	Stepped-wedge cluster RCT	ICUs	Y	2618-1434-1184	64.9	CAM-ICU and CAM	①②③④⑤
Faustino et al. (2022)	Brazil	RCT	ICUs	N	144-72-72	67.6	CAM-ICU	①②③④
Guo et al. (2016)	China	RCT	SICU	N	160-81-79	73.5	CAM-ICU	①②③⑤
Mitchell et al. (2017)	Australia	RCT	MICU and SICU	N	61-29-32	I: 52(32)a C: 60(21)a	CAM-ICU	①②③④
Rood et al. (2021)	Netherlands	RCT	ICUs	Y	1749-924-825	71.0	CAM-ICU	①②③⑤
Adineh et al. (2023)	Iran	RCT	ICUs	N	66-33-33	36.7	CAM-ICU	①②③④⑤
Álvarez et al. (2017)	Chile	RCT	MICU and SICU	N	140-70-70	84.1	CAM	①②③④⑤
Lin et al. (2024)	China	RCT	CSICU	N	80-40-40	61.0	CAM-ICU	①②③④⑤
Moon and Lee (2015)	Korea	RCT	MICU and SICU	N	123-60-63	69.7	CAM-ICU	①②③⑤
Contreras et al. (2021)	Colombia	RCT	SICU	N	81-40-41	I: ≥60[32(80%)] C: ≥60[33(80.5%)]	CAM-ICU	①②③④
Malik and Subramaniam (2021)	India	RCT	MICU and SICU	N	50-25-25	42.1	CAM-ICU	①②③④⑤
Dong et al. (2026)	China	Pilot RCT	CSICU	N	40-20-20	65.0	CAM-ICU	①②③⑤

ICU, Intensive Care Unit; RCT, randomized controlled trial; I, Intervention; C, Control; SICU, Surgical Intensive Care Unit; MICU, Medical ICU; NICU, Neurological ICU; CSICU, cardiovascular surgery ICU. ① Cognitive assessment and stimulation; ② Reorientation; ③ Management of sleep and environment; ④ Support from family and community; ⑤ Physical activity.

### 3.3 Risk-of-bias assessment

The risk-of-bias assessment identified six studies at low risk of bias, three at unclear risk, and four at high risk (see Figure 2). The main concerns were related to the difficulty of blinding participants and personnel in non-pharmacological interventions, incomplete reporting of allocation procedures in some trials, and potential concerns regarding outcome assessment or selective reporting in a small number of studies. Missing outcome data was generally judged to have a low risk of bias.

**Figure 2:**
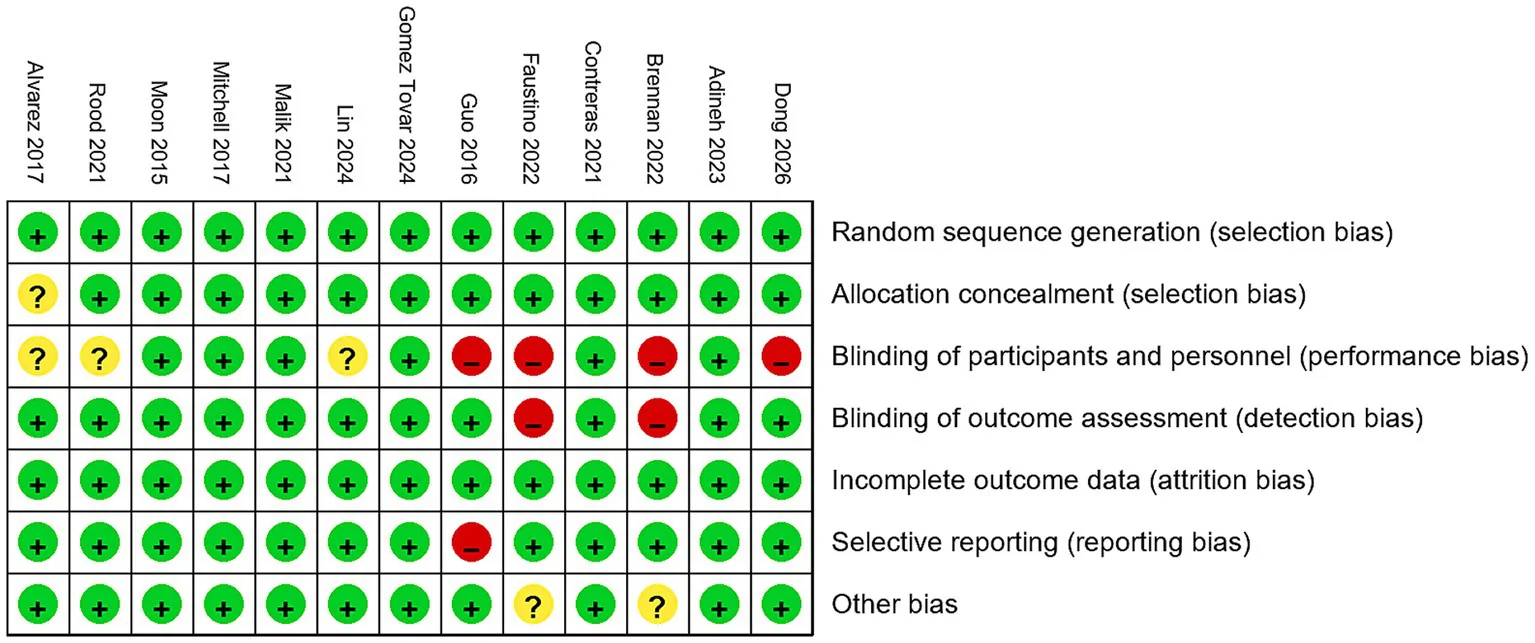
Risk-of-bias assessment of the included studies.

### 3.4 Synthesized findings

#### 3.4.1 Pooled analysis

The pooled analysis of 13 trials involving 5,525 ICU patients showed that multicomponent non-pharmacological interventions significantly reduced delirium incidence (RR 0.53, 95% CI [0.36, 0.76]; I^2^ = 72.5%) (Figure 3). Sensitivity analyses yielded consistent results. The effect remained significant after excluding the two cluster-randomized trials (11 trials; RR 0.45, 95% CI [0.30, 0.67]; I^2^ = 55.7%), the active-control trial by Dong et al. (12 trials; RR 0.55, 95% CI [0.38, 0.79]; I^2^ = 73.0%), and all three trials simultaneously (10 trials; RR 0.46, 95% CI [0.31, 0.70]; I^2^ = 57.0%).

**Figure 3:**
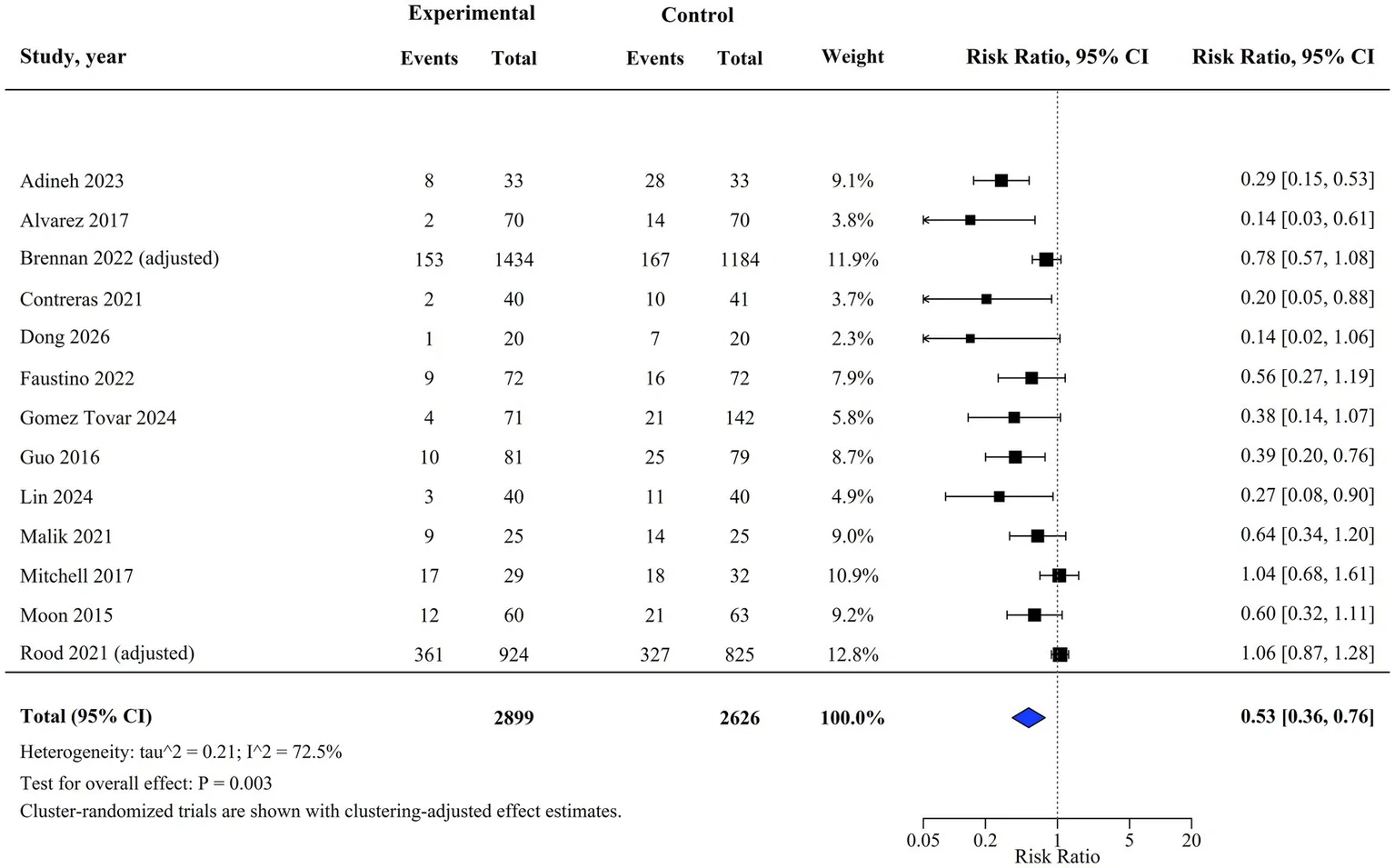
Forest plot of multicomponent non-pharmacological interventions versus control for delirium incidence.

No statistically significant effects were observed for delirium duration (MD -1.49 days, 95% CI [−3.67, 0.69]; I^2^ = 96.3%), ICU length of stay (MD -2.90 days, 95% CI [−6.83, 1.03]; I^2^ = 97.8%), hospital length of stay (MD -1.09 days, 95% CI [−4.27, 2.10]; I^2^ = 42.8%), ICU mortality (RR 0.77, 95% CI [0.48, 1.23]; I^2^ = 0%), 28-day mortality (RR 1.09, 95% CI [0.32, 3.71]; I^2^ = 0%), 90-day mortality (RR 1.13, 95% CI [0.38, 3.36]; I^2^ = 0%), or delirium-free days (MD 0.10 days, 95% CI [−0.71, 0.91]; I^2^ = 0%).

Hospital mortality was summarized narratively. Moon and Lee (2015) reported 4/60 deaths in the intervention group and 13/63 in the control group. Brennan et al. (2023) reported 146/1434 deaths after implementation and 145/1184 before implementation (adjusted RR 0.81, 95% CI 0.61–1.07).

#### 3.4.2 Subgroup analysis

Subgroup analyses revealed that component pattern B interventions significantly reduced delirium incidence (RR 0.43, 95% CI [0.23, 0.82]). The effects were not significant for component pattern A (RR 0.62, 95% CI [0.29, 1.33]) or component pattern C (RR 0.58, 95% CI [0.31, 1.07]). However, the formal test for subgroup differences was not significant (*p* = 0.604), and therefore the findings do not establish superiority of component pattern B (see Figure 4).

**Figure 4:**
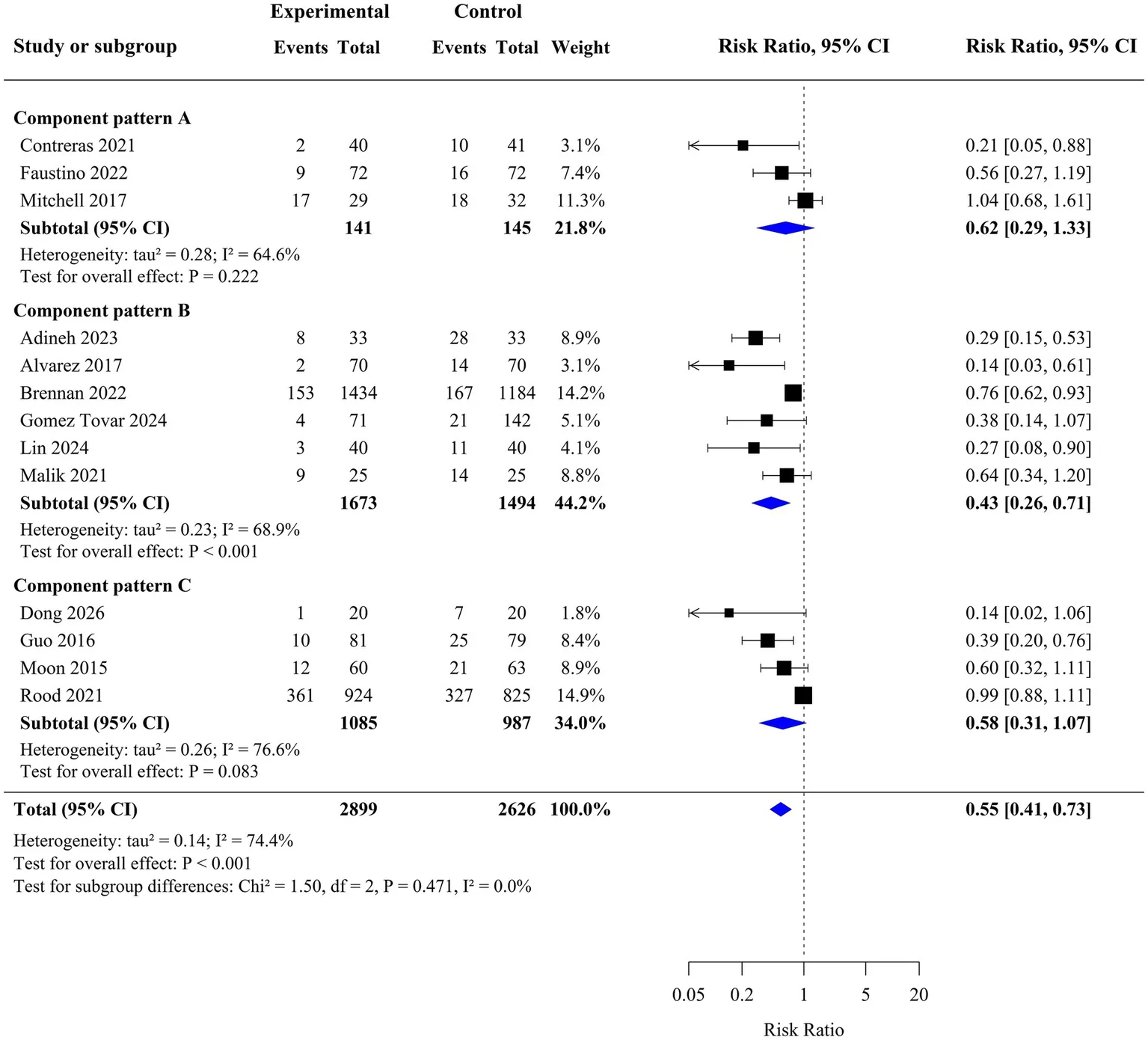
Subgroup analysis of delirium incidence according to intervention component patterns.

#### 3.4.3 Publication bias

Publication bias was assessed for the primary outcome of delirium incidence across the 13 included studies. Visual inspection of the funnel plot indicated an asymmetric distribution (see Figure 5), and Egger random-effects regression: slope −2.756 (95% CI -3.794 to −1.718), *p* < 0.001. These findings suggest that potential publication bias could not be excluded.

**Figure 5:**
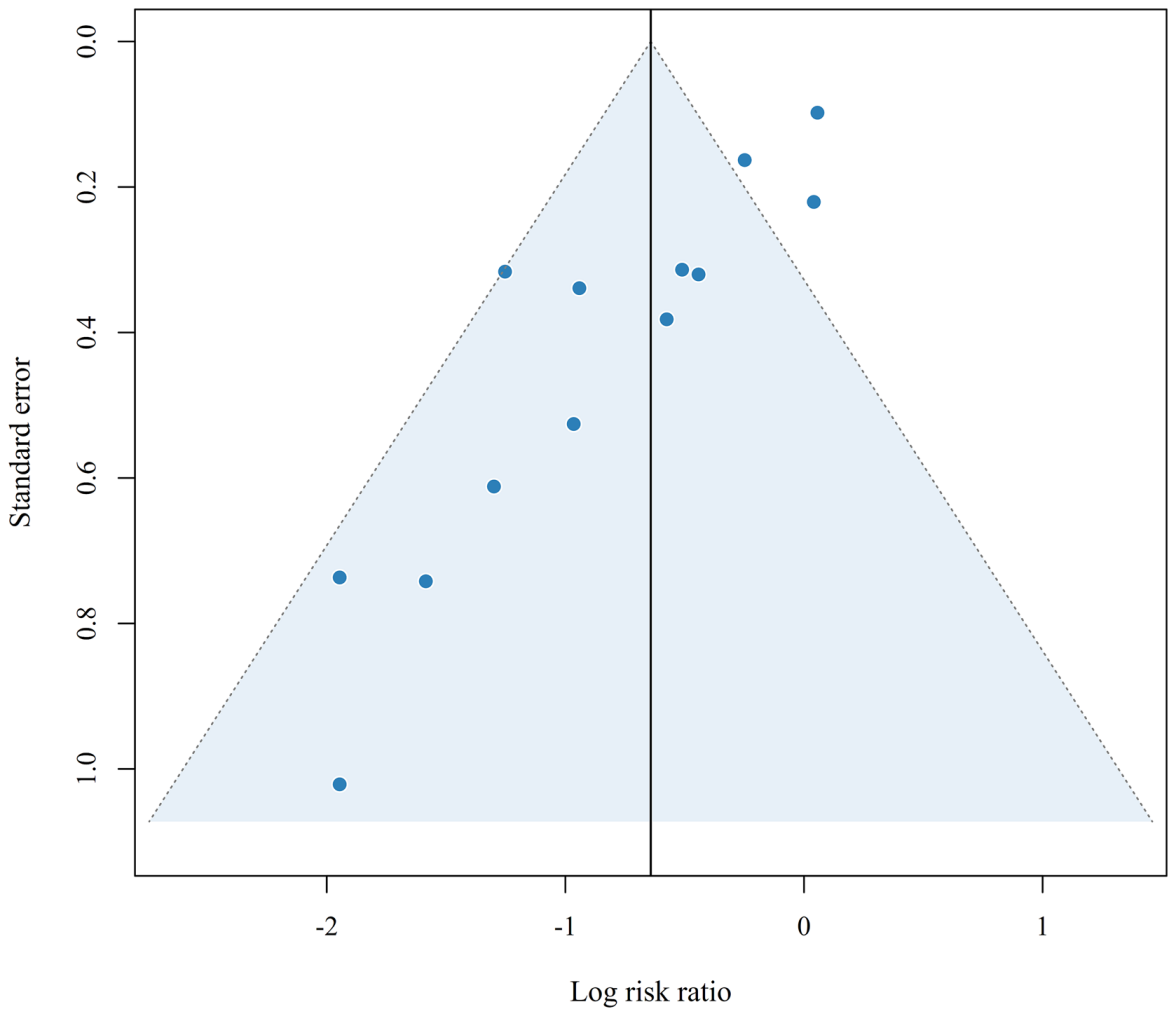
Funnel plot of delirium incidence.

#### 3.4.4 Certainty of evidence

The certainty of evidence for delirium incidence was rated as low because of risk of bias and substantial inconsistency. Certainty for the secondary outcomes ranged from low to very low, mainly because of risk of bias, inconsistency, and imprecision (Table 4).

**Table 4:** Summary of findings and certainty of evidence for primary and secondary outcomes.

Outcome	No. of participants (studies)	Effect estimate	Certainty of evidence (GRADE)	Main reasons for downgrading/Comments
Delirium incidence	5,525 (13 RCTs)	RR 0.53 (95% CI 0.36 to 0.76)	Low ⊕ ⊕ ◯◯	Downgraded for risk of bias and substantial inconsistency.
Delirium duration	2,443 (7 RCTs)	MD −1.49 days (95% CI −3.67 to 0.69)	Very low ⊕◯◯◯	Downgraded for risk of bias and very serious inconsistency.
ICU length of stay	5,244 (10 RCTs)	MD −2.90 days (95% CI −6.83 to 1.03)	Very low ⊕◯◯◯	Downgraded for risk of bias and very serious inconsistency.
Hospital length of stay	4,802 (7 RCTs)	MD −1.09 days (95% CI −4.27 to 2.10)	Low ⊕ ⊕ ◯◯	Downgraded for risk of bias, inconsistency, and imprecision.
ICU mortality	3,049 (5 RCTs)	RR 0.77 (95% CI 0.48 to 1.23)	Low ⊕ ⊕ ◯◯	Downgraded for risk of bias and imprecision.
Hospital mortality	2,741 (2 RCTs)	Not pooled; summarized narratively.	Very low ⊕◯◯◯	Downgraded for risk of bias and very serious imprecision.
28-day mortality	1,799 (2 RCTs)	RR 1.09 (95% CI 0.32 to 3.71)	Very low ⊕◯◯◯	Downgraded for risk of bias and limited evidence from few studies.
90-day mortality	1,893 (2 RCTs)	RR 1.13 (95% CI 0.38 to 3.36)	Very low ⊕◯◯◯	Downgraded for risk of bias and limited evidence from few studies.
Delirium-free days	2,812 (3 RCTs)	MD 0.10 days (95% CI −0.71 to 0.91)	Low ⊕ ⊕ ◯◯	Downgraded for risk of bias and limited number of studies.

## 4 Discussion

The objective of this meta-analysis was to evaluate the effectiveness of non-pharmacological interventions for ICU delirium and identify specific intervention combinations. This updated study included 13 randomized controlled trials with 5,525 participants. Compared with control conditions, multicomponent non-pharmacological interventions significantly reduced delirium incidence (RR 0.53, 95% CI [0.36, 0.76]). This result remained significant in the sensitivity analyses. However, no significant effects were found for delirium duration, ICU or hospital length of stay, delirium-free days, or mortality. These findings are consistent with previous studies (Chen et al., 2022; Deng et al., 2020; Matsuura et al., 2023), providing further support for the potential preventive effect of multicomponent non-pharmacological interventions on delirium incidence.

Unlike Chen’s et al. (2022) study, which compared single-component and multicomponent non-pharmacological interventions, our meta-analysis explored specific multicomponent patterns for preventing ICU delirium. Matsuura et al. (2023) proposed that the “sleep promotion-cognitive stimulation-early activity-pain control-assessment” combination was the most effective but recommended the “sleep promotion-cognitive stimulation” combination because of practical workload and time constraints. Their study included 11 studies, of which only three were RCTs. In contrast, our updated meta-analysis incorporated 13 RCTs, providing updated randomized evidence.

Recent pharmacological evidence also illustrates the difficulty of defining an optimal strategy for delirium prevention. In a Bayesian network meta-analysis of patients undergoing cardiac surgery, dexmedetomidine combined with melatonin ranked as the most promising pharmacological option, although the certainty of evidence was low (Queiroz et al., 2026). Although their review addressed pharmacological prevention in a surgical population, it is relevant to the present study because both reviews point to the same practical challenge: delirium prevention often involves combined or multidomain approaches, yet the most effective composition remains uncertain. These findings reinforce the need for future trials to define intervention components clearly, report implementation fidelity, and identify which strategies are most suitable for specific clinical populations or ICU settings.

Although debates persist regarding whether multicomponent non-pharmacological interventions can reduce delirium duration and ICU length of stay (Chen et al., 2022; Wang et al., 2024), the updated pooled estimates for these outcomes were not statistically significant. The heterogeneity observed across outcomes likely reflects the clinical and methodological complexity of ICU delirium prevention rather than a single source of variation. The included trials differed in patient vulnerability, with age, comorbidity burden, illness severity, and baseline delirium risk potentially influencing responsiveness to prevention. ICU context may also have modified the observed effects: surgical and cardiac surgical ICUs involve perioperative stress and structured recovery pathways, whereas medical and mixed ICUs often include more heterogeneous critical illness, sedation exposure, mechanical ventilation, and organ support. Outcome detection may also have varied because CAM and CAM-ICU, differ in structure, timing, and diagnostic performance. Finally, intervention intensity, implementation fidelity, and comparator care varied across trials. Thus, the pooled estimates should be interpreted as the average effects of related multicomponent non-pharmacological strategies rather than the effects of a single standardized protocol. The risk-of-bias assessment identified six studies at low risk of bias, three at unclear risk, and four at high risk, with the main concerns related to blinding.

This study categorized multicomponent non-pharmacological interventions into five core components (cognitive assessment and stimulation, reorientation, sleep and environmental management, family support, and physical activity) and grouped the included studies into three combinations: component pattern A (lacking physical activity), component pattern B (including all five components), and component pattern C (lacking family support). Meta-analysis of these combinations showed different point estimates, but the formal test for subgroup differences was not significant.

Subgroup analysis indicated that component pattern B, which included cognitive assessment and stimulation, reorientation, sleep and environmental management, family support, and physical activity, was associated with reduced delirium incidence. However, subgroup-specific significance does not demonstrate a difference between intervention combinations. The formal interaction test was not significant, and each subgroup contained few studies. Therefore, this finding should be considered exploratory. In the high-risk and high-pressure ICU environment, nurses remain central to intervention delivery through close monitoring and timely adjustment. Future research should standardize intervention timing, frequency, intensity, and fidelity and should directly evaluate the incremental value of family support and physical activity.

Physical activity and family support remain clinically plausible components of effective interventions and are consistent with the PADIS guideline (Devlin et al., 2018). However, their independent or incremental effects cannot be isolated reliably from the present subgroup data because intervention components were delivered simultaneously and the component combinations were not randomized comparisons.

These findings have practical implications for ICU delirium prevention. Rather than supporting a single fixed bundle, the results favor a structured but adaptable approach in which core non-pharmacological components are selected according to patient risk, ICU workflow, and available resources. Synthesizing the intervention components may provide a practical starting point for translating delirium-prevention principles into ICU bedside care, but implementation should focus not only on whether these components are included, but also on whether they are delivered consistently and early enough during ICU care.

### 4.1 Limitations

This updated review included 13 randomized controlled trials involving 5,525 participants, but several limitations should be considered. The interventions varied in timing, frequency, intensity, and implementation fidelity, which may have contributed to heterogeneity. Blinding was often impracticable because of the nature of non-pharmacological interventions, and several trials had relatively small samples. Component classification required clinical judgment because intervention descriptions varied across studies; therefore, the subgroup analyses should be considered exploratory. Funnel-plot asymmetry and Egger’s test suggested small-study effects, although publication bias could not be distinguished from other possible causes of asymmetry. For continuous outcomes, differences in reporting formats, including the use of medians and interquartile ranges, may have reduced comparability across studies. Restriction to English-language publications may also have introduced language bias. These limitations were reflected in the GRADE assessment, which indicated low certainty for delirium incidence and low to very low certainty for the secondary outcomes. Accordingly, the findings suggest that multicomponent interventions may reduce delirium incidence, but their effects on secondary outcomes and the optimal combination of components remain uncertain.

## 5 Conclusion

This study demonstrates that multicomponent non-pharmacological interventions may reduce the incidence of delirium in ICU patients, although the certainty of evidence was low. Although the component pattern including cognitive assessment and stimulation, reorientation, sleep and environmental management, family support, and physical activity showed a significant within-subgroup pooled effect, the interaction test did not establish superiority over other component combinations. Evidence regarding delirium duration, length of stay, and mortality remained inconclusive, with certainty ranging from low to very low. Further multicenter randomized trials with standardized intervention content, timing, intensity, and implementation fidelity are required.

## Data Availability

The original contributions presented in the study are included in the article/Supplementary material, further inquiries can be directed to the corresponding author.
