# Supplementary material for: Multicomponent non-pharmacological interventions for preventing delirium in critically ill patients: a systematic review and meta-analysis of randomized controlled trials
Source: Front Neurosci. 2026 Sep 24;20:1927572. doi: 10.3389/fnins.2026.1927572 (PMC13647169; doi:10.3389/fnins.2026.1927572)
Supplement: Supplementary file 1 [file Supplementary_file_1.docx]

**Supplementary Fig.1**

*Delirium duration（days）*

**Supplementary Fig.2**

*Length of ICU stay (days)*

**Supplementary Fig.3**

*ICU mortality*

**Supplementary Fig.4**

*Length of hospital stay(days)*

**Supplementary Fig.5**

*28-day mortality*

**Supplementary Fig.6**

*90-day mortality*

**Supplementary Fig.7**

*Hypoactive delirium*

**Supplementary Fig.8**

*Delirium-free days*

**Supplementary Fig.9**

*Subgroup delirium duration (days)*

**Supplementary Fig.10**

*Subgroup length of ICU stay (days)*

**Supplementary Fig.11**

*Multi-group B(①-⑤) ICU mortality*

**Supplementary Fig.12**

*Multi-group B(①-⑤) length of hospital stay(days)*
